# Supplementary material for: Salvianolic Acid B in Microemulsion Formulation Provided Sufficient Hydration for Dry Skin and Ameliorated the Severity of Imiquimod-induced Psoriasis-like Dermatitis in Mice
Source: Pharmaceutics. 2020 May 17;12(5):457. doi: 10.3390/pharmaceutics12050457 (PMC7284938; doi:10.3390/pharmaceutics12050457)
Supplement: Supplementary file 1 [file pharmaceutics-12-00457-s001.pdf]

pharmaceutics

MDPI

Supplementary Materials: Salvianolic Acid B in Microemulsion Formulation Provided Sufficient Hydration for Dry Skin and Ameliorated the Severity of Imiquimod-induced Psoriasis-like Dermatitis in Mice

Jiun-Wen Guo, Yu-Pin Cheng, Chih-Yi Liu, Haw-Yueh Thong, Chi-Jung Huang, Yang Lo, Chen-Yu Wu and Shiou-Hwa Jee

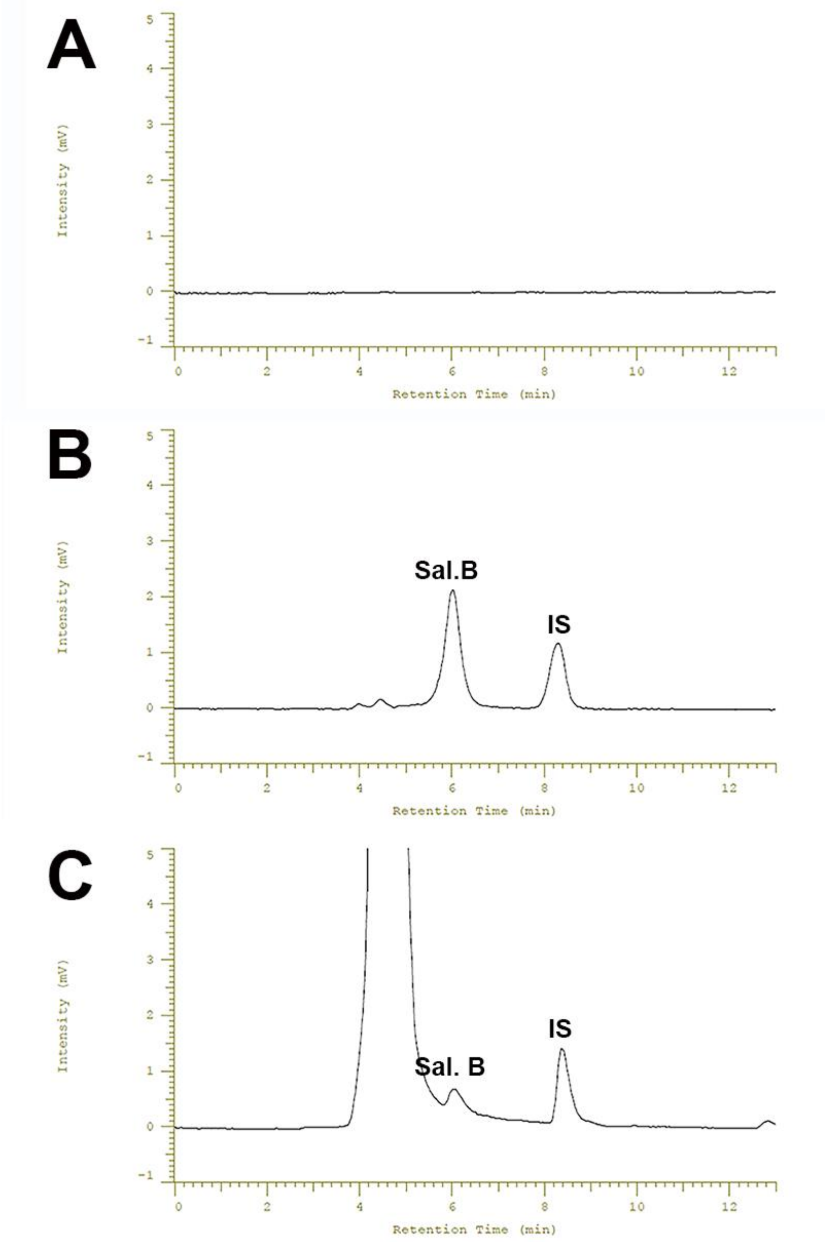

Figure S1. Representative high-performance liquid chromatography of Sal. B and chloramphenicol. (**A**) blank (**B**) standard with Sal. B (1 μg/mL) and internal standard (chloramphenicol) (**C**) receiver concentration at 1 h after a 200 μL of Sal. B (300 μg/mL) was added to the donor cell. The retention time of Sal. B and chloramphenicol (IS) was 6.0 ± 0.1 min and 8.3 ± 0.1 min, respectively. Sal. B, Salvianolic acid B; IS, internal standard.

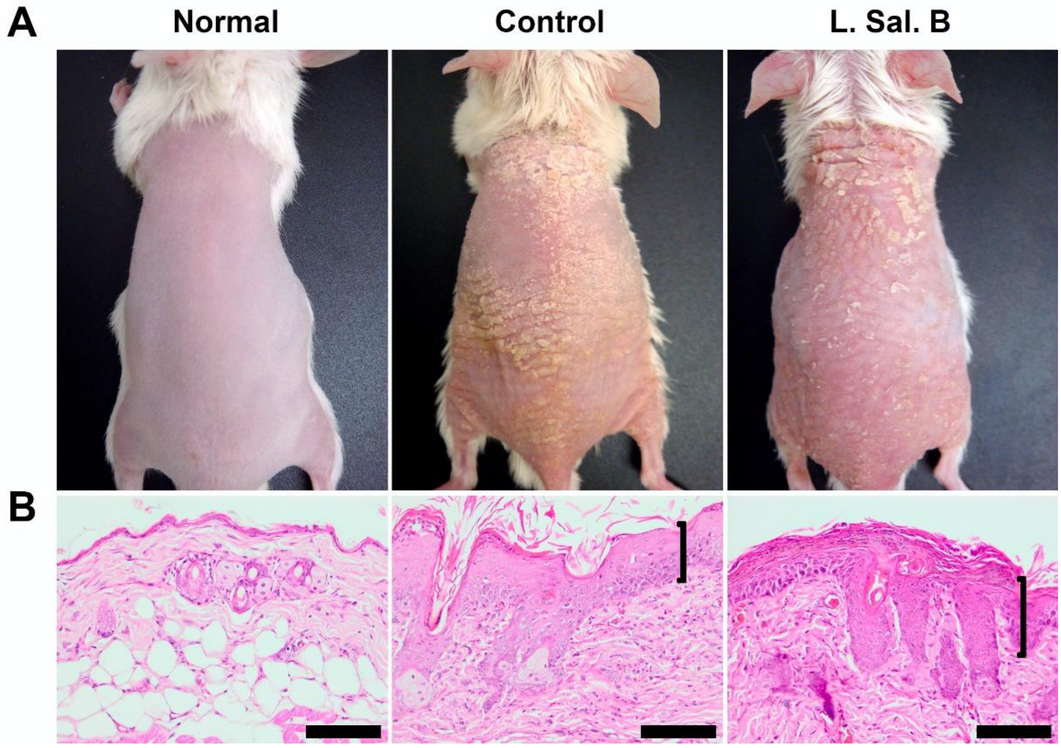

Figure S2. Low dose of Sal. B/formulation A did not improve psoriasis-like dermatitis. The low dosage of Sal. B (100 μg/mL) in formulation A treatment group showed no significant difference in clinical and pathological features of severity index compare to the control group. Therefore, the 300 μg/mL Sal. B in formulation A was chosen as the optimal dose for further study. Scale bar = 100 μm. Brackets indicate thickness of epidermis.
